# Dataset showing the abundance and distribution of benthic foraminifera in relation to marine sediment parameters from western Arabian Gulf

**DOI:** 10.1016/j.dib.2019.105014

**Published:** 2019-12-17

**Authors:** Abduljamiu O. Amao, Mohammad A. Qurban, Michael A. Kaminski, Thadickal V. Joydas, Ponnambalam K. Manikandan, Fabrizio Frontalini

**Affiliations:** a Center for Integrative Petroleum Research (CIPR), College of Petroleum Engineering and Geosciences (CPG), King Fahd University of Petroleum and Minerals (KFUPM), Dhahran 31261, Saudi Arabia; b Center for Environment and Water, Research Institute, King Fahd University of Petroleum and Minerals (KFUPM), Dhahran 31261, Saudi Arabia; c Geosciences Department, College of Petroleum Engineering and Geosciences, King Fahd University of Petroleum and Minerals (KFUPM), Dhahran 31261, Saudi Arabia; d Dipartimento di Scienze Pure e Applicate (DiSPeA), Università degli Studi di Urbino “Carlo Bo”, Campus Scientifico, Località Crocicchia, 61029 Urbino, Italy

**Keywords:** Benthic foraminifera, Endemic species, Pollution, PTE, Heavy metals, Trace metals, Arabian Gulf

## Abstract

This dataset supports the paper entitled “A baseline investigation of benthic foraminifera in relation to marine sediments parameters in western parts of the Arabian Gulf”. Duplicate sediment samples (sets A and B) were collected from 30 stations in an area covering approximately 25000 km^2^ in the offshore northern Arabian Gulf, using a van Veen grab (0.1 m^2^ area) and the top 1 cm was analysed for living benthic foraminifera. A set of samples was devoted to foraminiferal analysis while the other, set B, for sediment analyses. *In situ* hydrographical parameters such as temperature, salinity, pH, turbidity and DO were measured at surface waters. The top 1 cm was subsampled for foraminiferal analyses from the grab and preserved using 70% ethanol with Rose-Bengal stain. Potentially Toxic Elements (PTE) levels in sediment and grain size distributions were analysed. The dataset is expected to provide a baseline for PTE levels in sediment, benthic foraminiferal communities, and identify endemic species adapted to extremes of temperature and saline conditions typical of the Gulf. It can also be used by environmental managers, micropaleotologists, students in environmental/geology/marine science as reference background conditions based on sediment toxicity and benthic community information in revising environmental guidelines in the region. Data from this study suggest that PTEs are within the range of background values, and the sediments support highly diversified and stable benthic foraminiferal communities adapted to the unique environmental conditions in the Gulf. To date, this dataset documents the highest number of living benthic foraminifera species reported from the Gulf, and the most diverse living community compared to all previous studies. It also provides evidence for the full recovery of areas impacted during the 1991 Gulf oil spill which is evident by the diverse and flourishing assemblages of living benthic foraminifera documented.

**Table:** Specifications Table

Subject	Environmental Science
Specific subject area	Management, Monitoring, Policy and Law/Pollution
Type of data	Tables, Figures and Appendices
How data were acquired	Dataset was acquired through manual sediment sample picking, YSI Multi Parameter Water Quality Sonde Model 6600 V2 (temperature, salinity, pH, turbidity and DO), Scanning Electron Microscope (SEM micrographs), Coupled Plasma Optical Emission Spectrometry (for elements As, Al, Fe Cd, Co, Cr, Cu, V, Ni, Hg, Pb, and Zn), PAST v3.12 software (statistical summary).
Data format	• Raw • Analysed
Parameters for data collection	Duplicate sediment samples (sets A and B) were collected from 30 stations in an area covering approximately 25000 km^2^ in offshore northern Arabian Gulf, using a van Veen grab (0.1 m^2^ area) and the top 1 cm was analysed for living benthic foraminifera and Potentially Toxic Elements (PTEs).
Description of data collection	For the foraminiferal analyses, the top 1 cm was subsampled from the grab and preserved using 70% ethanol with Rose-Bengal stain in 100 ml plastic jars. Samples were preserved in Rose Bengal – Ethanol solution to prevent protoplasm decay and to make the separation of the living (stained) and dead (non- stained) easier. All jars were labelled in the field, on the side and lid with relevant information such as date, geographical coordinates, depth of sampling, and salinity. Preserved samples were held in the laboratory for two weeks to allow for proper staining, after which they were wet-sieved through a 63 μm mesh sieve, dried, and later split into equal aliquots using a micro-splitter to generate subsamples with approximately 300 benthic foraminiferal tests. Rose Bengal stained specimens were picked quantitatively from the >125 μm fraction to exclude juveniles. Living benthic foraminifera were largely used to infer autochthonous origin of species and their interactions with biotic and abiotic components of their immediate environment.
Data source location	King Fahd University of Petroleum and Minerals, Dhahran, Saudi Arabia and The Arabian Gulf (Middle East)
Related research article	[1] Amao, A. O., Qurban, M. A., Kaminski, M. A., Joydas, T. V., Manikandan, P. K., & Frontalini, F. (2019). A baseline investigation of benthic foraminifera in relation to marine sediments parameters in western parts of the Arabian Gulf. *Marine Pollution Bulletin*, *146*, 751–766. https://doi.org/10.1016/j.marpolbul.2019.06.072

**Table:** 

**Value of the Data** • The dataset documents the distribution and abundance of living benthic foraminifera in relation to Potentially Toxic Elements (PTEs) • It is expected to provide a baseline for PTE levels in sediment, benthic foraminiferal communities, and identify endemic species adapted to extremes of temperature and saline conditions typical of the Gulf. • To date, this data set documents the highest number of living benthic foraminifera species reported from the Gulf, and the most diverse living community compared to all previous studies. • It also provides evidence for the full recovery of areas impacted during the 1991 Gulf oil spill which is evident by the diverse and flourishing assemblages of living benthic foraminifera documented.

## 1 Data

**Fig. 1:**
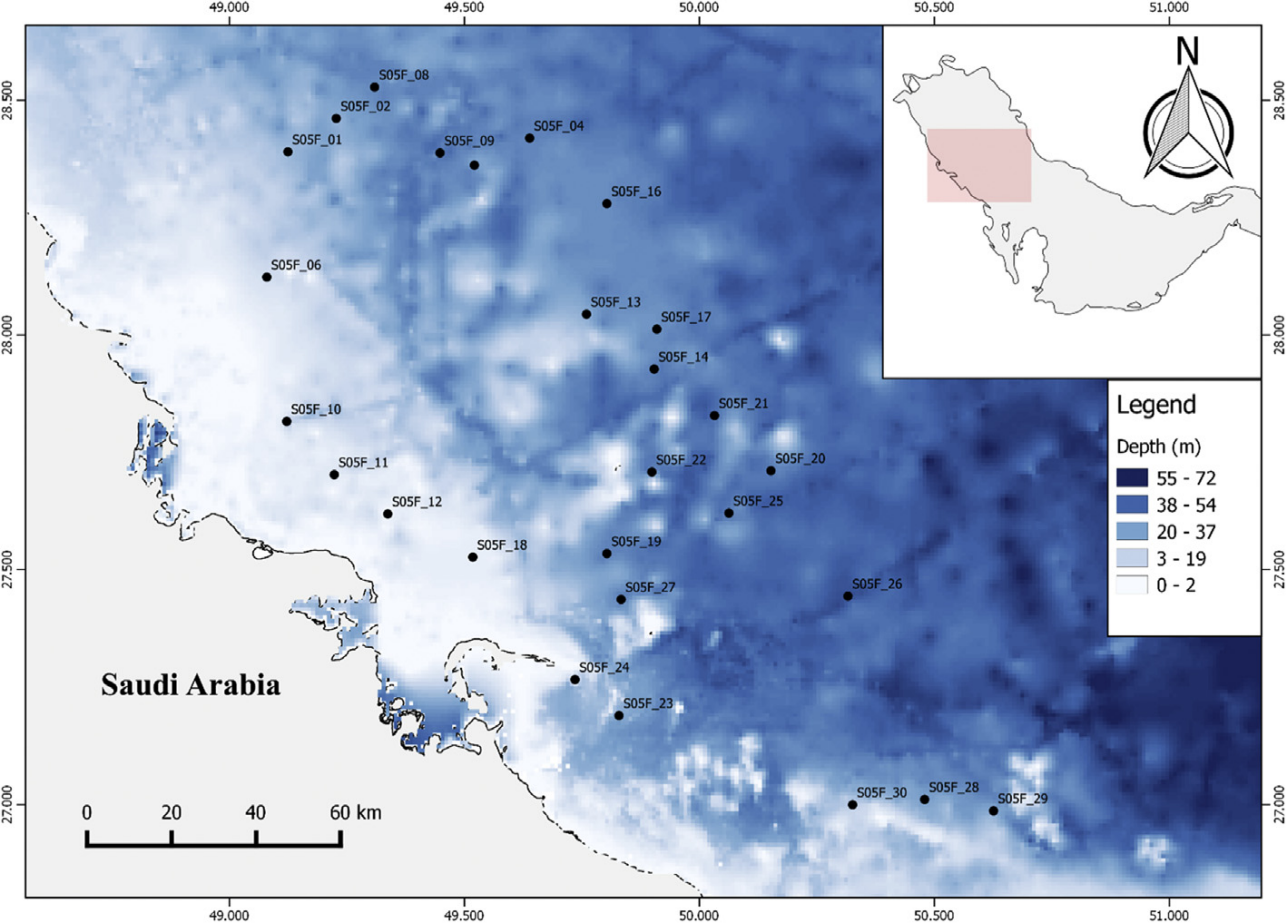
Sampled area with a detailed bathymetry profile. Coordinates (DMS) are provided in Appendix 1-1.

**Fig. 2:**
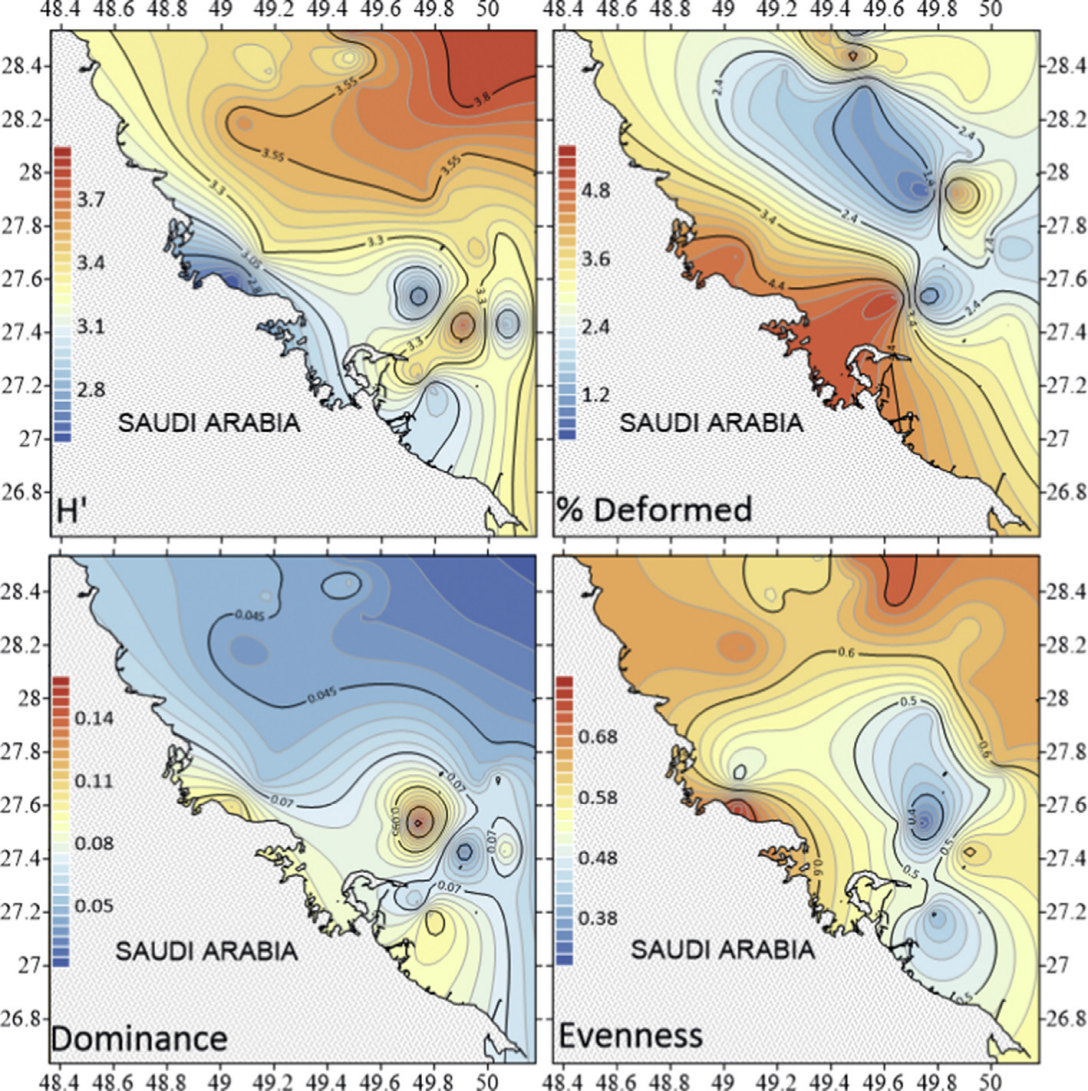
Gradients for Shannon Weiner Index (H′), Percentage Deformity, Dominance and evenness for the sampled stations Appendix 1-2.

**Fig. 3:**
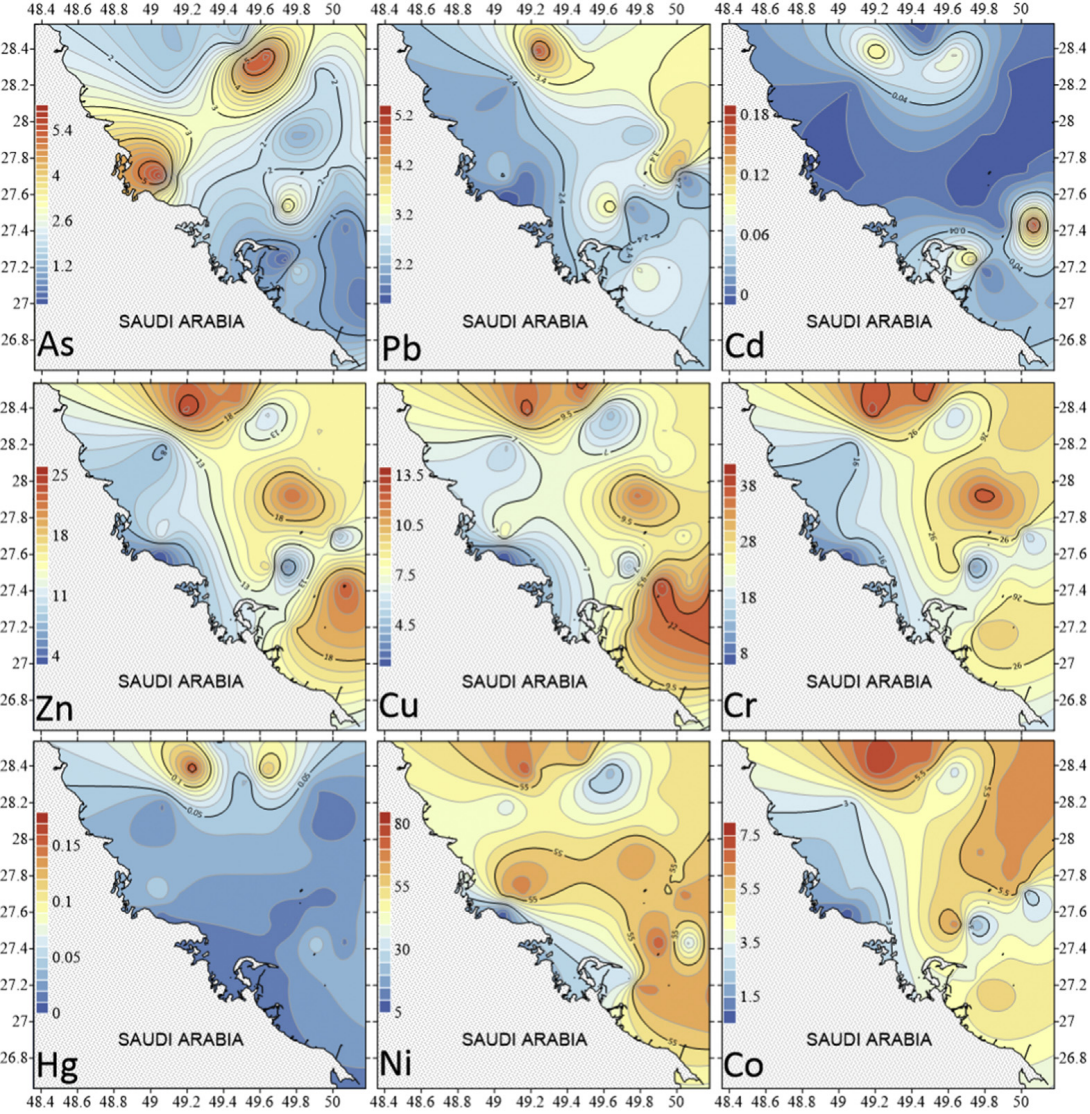
Distribution of concentration gradients for of selected PTE in the sample area. There is generally higher PTE concentration in the deeper offshore direction which corresponds to areas with higher mud content. Appendix 1-3 documents the values compared to proposed Gulf levels and a modified Norwegian guideline for marine sediments. Proposed Gulf levels except for Ni are significantly lower.

**Fig. 4:**
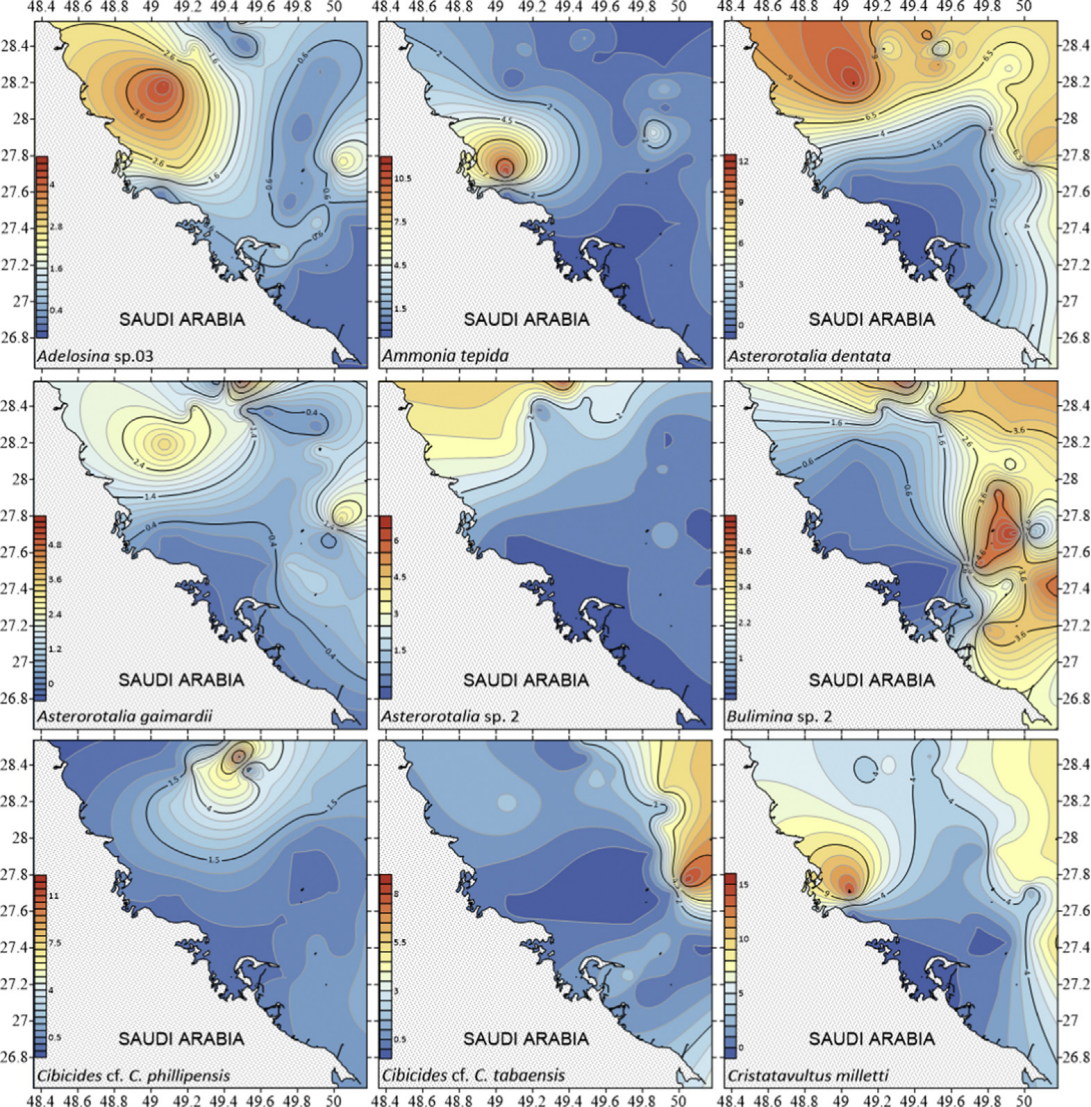
Spatial distribution of the most abundant species identified Species were selected based on relative abundance ≥3% from each station sampled area. Species arrange alphabetically *Adelosina* sp 3 – *Cristatavultus milletti*. Raw counts of benthic foraminifera collected from each station with their corresponding detailed taxonomy are documented in Appendix 1-4 and standardised counts are presented in Appendix 1-5.

**Fig. 5:**
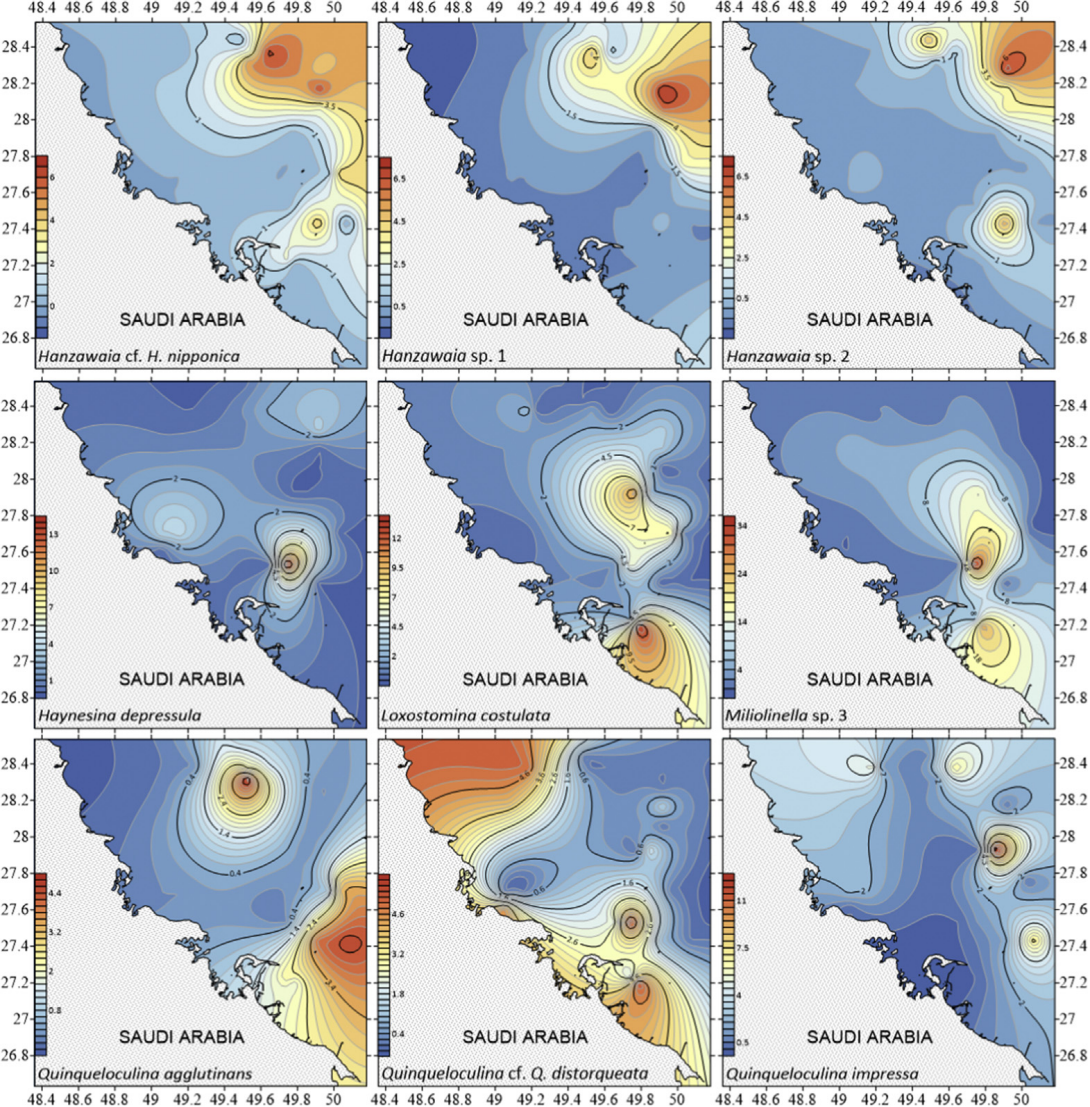
Spatial distribution of the most abundant species identified Species were selected based on relative abundance ≥3% from each station sampled area. Species arrange alphabetically *Hanzawaia* cf. *H. nipponica* – *Quinqueloculina impressa*.

**Fig. 6:**
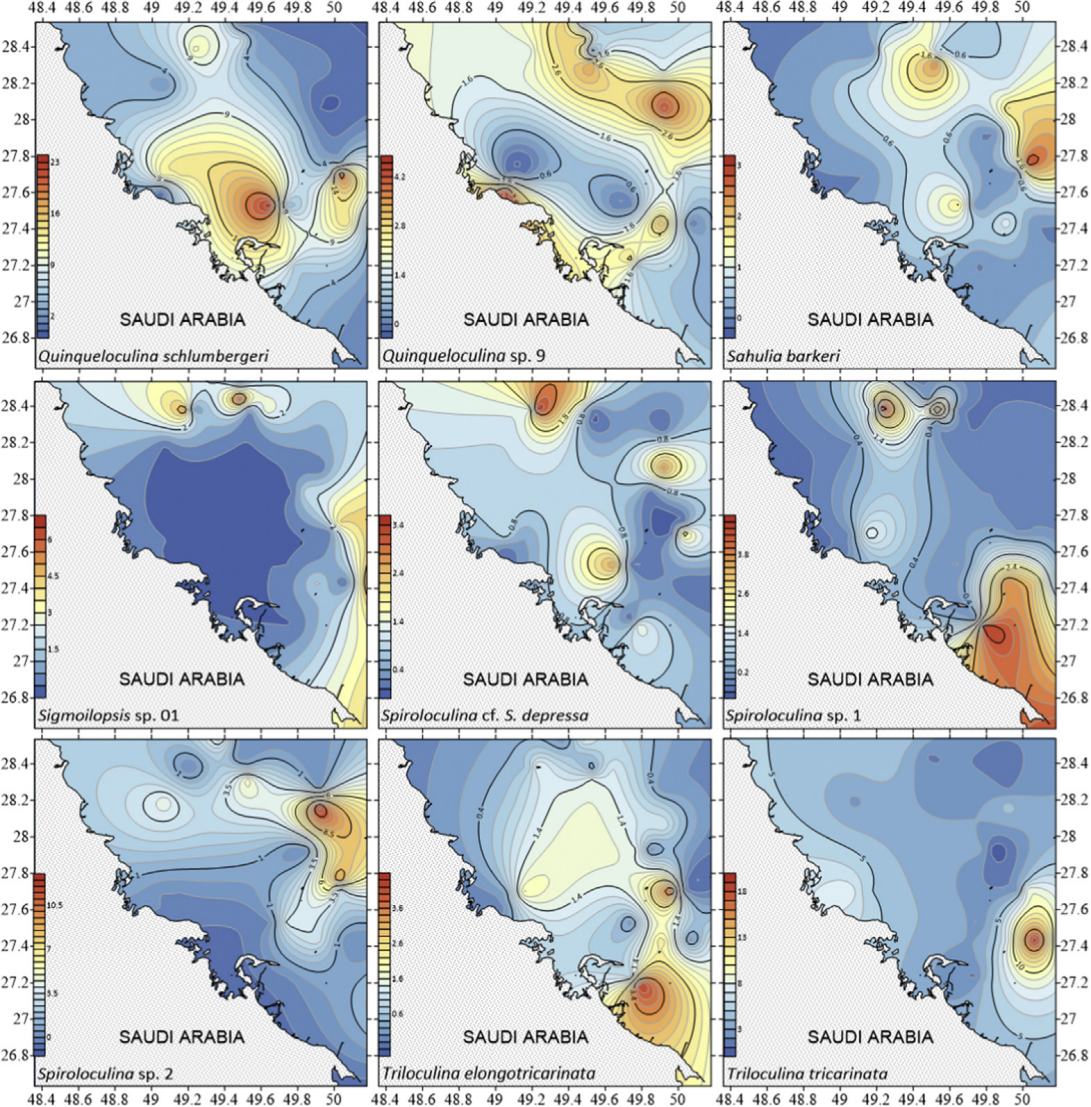
Spatial distribution of the most abundant species identified Species were selected based on relative abundance ≥3% from each station sampled area. Species arrange alphabetically *Quinqueloculina schlumbergeri* – *Triloculina tricarinata*.

**Fig. 7:**
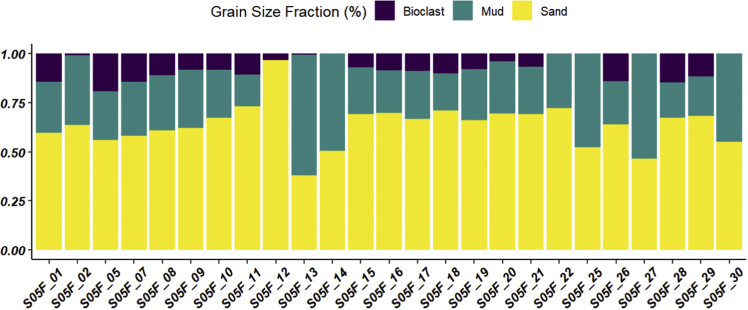
Grain size distribution along the sampled station, details including textural classes are presented in Appendix 1-6.

The dataset contains raw and standardised benthic foraminifera counts, measured water and sediment parameters collected through manual sediment sample picking, water quality multiprobe and coupled plasma optical emission spectroscopy measurements respectively. Fig. 1 shows the area where the samples were collected while the location coordinates for the sampled stations are attached in Appendix 1-1. Fig. 2 and Appendix 1-2 provides spatial representation of diversity indices that were calculated from raw foraminifera counts. Measured elemental concentration are summarised in Fig. 3 while the raw data are available in Appendix 1-3. Most abundant species identified, which were selected based on relative abundance ≥3% from each station in the sampled area are spatially presented Fig. 4, Fig. 5, Fig. 6. The documented raw counts and standardised counts of each species picked are attached in Appendix 1-4 and Appendix 1-5 respectively. Variation in grain sizes among the sample station is presented in Fig. 7 while the raw data is attached as Appendix 1-6. Table 1 (Appendix 1-7.) summarises water column physicochemical parameters.

**Table 1:** Summary of measured environmental variables from the water column, details are documented in Appendix 1-7. The deepest station sampled was S05F_25 (60.2 m) while the shallowest one was S05F_12 (5.7 m). The maximum and minimum temperature recorded were S05F_12 (33.7) and S05F_06 (29.7) oC respectively. The most saline station was S05F_10 (42.9) while the least saline was S05F_04 (39.6). The highest recorded pH was at S05F_14 (8.3) while the lowest was at S05F_03 (8.0). Among all the stations sampled, S05F_24 (2.1) was the most turbid while S05F_30 (0.8) was the least turbid. Dissolved oxygen was highest at S05F_21 (6.7) and lowest at S05F_11 (5.3).

Physical Parameters	Unit	Mean	SD
Depth	m	37.7	16.1
Temperature	°C	32.1	0.8
Salinity	psu	40.5	0.9
pH		8.1	0.1
Turbidity	FTU	1.4	0.4
DO	mg/L	6.0	0.3

## 2 Experimental design, materials, and methods

Duplicate sediment samples (sets A and B) were collected from 30 stations in an area covering approximately 25000 km^2^ in the offshore northern Arabian Gulf, using a van Veen grab (0.1 m^2^ area) and the top 1cm was analysed for living benthic foraminifera. The samples were collected during July–August 2014, in duplicates (sets A & B). A sample was devoted to foraminiferal analysis while the other, set B, for sediment analyses. In situ hydrographical parameters such as temperature, salinity, pH, turbidity and DO were measured at surface waters using a YSI Multiprobe Environmental Monitoring System (YSI Multi Parameter Water Quality Sonde, Model 6600 V2). PTE levels in sediment were measured according to modified US EPA 3050 preparation protocol. While concentration of selected metals including As, Al, Fe Cd, Co, Cr, Cu, V, Ni, Hg, Pb, and Zn were determined following the US EPA 6010 method, using Inductively Coupled Plasma Optical Emission Spectrometry (ICP-OES) at the Center for Environment and Water at the Research Institute at KFUPM. Grain-size analysis was carried out using wet and dry sieving techniques while for foraminiferal analyses, the top 2 cm was subsampled from the grab and preserved using 70% ethanol with Rose-Bengal stain in 100 ml plastic jars. Samples were preserved in Rose Bengal – Ethanol solution to prevent protoplasm decay and to make the separation of the living (stained) and dead (non-stained) easier. The identification of foraminifera was conducted based on the works detailed in Amao et al. [1]. Although no comprehensive taxonomical guide to the foraminifera of the Gulf region yet exists, one of the broader objectives of this data set is to make a taxonomical reference collection of Gulf Foraminifera. The faunal reference microslides are currently housed in the author's collection at KFUPM. These will be permanently archived in the European Micropaleontological Reference Center at Micropress Europe in Kraków (Poland). World Register of Marine Species collections were used to verify the validity of taxonomic names and groups [2]. Raw counts of living foraminifera at each station were used to calculate species richness (S), dominance (D), Shannon-Wiener (H′), and evenness (êH/S) indices using the PAST v3.12 software package [3].
